# Exploring digital support for the student transition to university through questionable concepts

**DOI:** 10.1007/s00779-021-01570-z

**Published:** 2021-05-04

**Authors:** Lisa Thomas, John Vines, Pam Briggs

**Affiliations:** 1 Psychology Department, Northumbria University, Newcastle upon Tyne, UK; 2 Institute for Design Informatics, University of Edinburgh, Edinburgh, UK

**Keywords:** Life transitions, University students, Questionable concepts

## Abstract

New students face challenges when they make the transition from school to university. Existing digital technologies used during this transition can sometimes increase the stressors associated with change. In order to explore ways forward for technology design in this space, we developed a brochure of questionable concepts. The concepts were grounded in findings of our prior research, yet were also intended to act as provocations to promote discussion in workshops involving 32 first year university students. Our analysis of workshop discussions documents the diverse issues students face around social bonding, their home environment, and their academic performance. Our findings challenge assumptions made in prior work about the ease of transition to university. We demonstrate how questionable concepts can play an important role in prompting ‘safe’ conversations around stressful life events for adolescents.

## Introduction

There are significant challenges for new students making the transition to university. Those who have moved to a new country or city may be fearful about making new friends or ‘fitting in’ (particularly those who are the first person in their family to attend university, rural students or international students) [1, 2], while those who remain at home can experience problems of integration with their new peers [3]. In the weeks leading up to, and on arrival at university, many students become anxious about how well they will succeed and experience loneliness, depression and social anxiety in many forms. It is perhaps not surprising that more than one-third of first year university students in eight industrialised countries around the globe report symptoms consistent with a diagnosable mental health disorder [4]. Often, such problems are associated with a vicious circle wherein poor mental health leads to poor academic performance and in turn high anxiety [5, 6].

Internationally, various programmes are being put in place at universities to support students during this transition period. Many of these are reliant upon digital services to help students make new friends [7], adjust to new cultures [8] as well as to acquaint them with the staff and services of the university [9]. In the US, work has demonstrated the importance of peer networks for new students, and their absence is associated not only with poor course performance, but drop-out from the course entirely [10, 11]. As such, social media (SM) can facilitate relationship building [12, 13] as well as online access to other students and staff which, in turn, can predict successful integration into university life [14]. However, work also demonstrates that SM use during the transition to university can cause problems, for example when social comparisons with peers makes students feel inadequate [15] or when personal information bleeds from one digital space to another in a process known as ‘context collapse’ [16]. In short, while student mental health problems, including depression and anxiety, are commonplace [e.g. 17], it appears that SM and other online platforms which intend to ameliorate these do not always help. We also know that some institutions make attempts to broker friendships between students early on [8], but we do not know if these systems work, nor what students would like to see in terms of digital support as they prepare for their early days and weeks at university.

In this paper, we report on a qualitative study where we set out to explore with UK-based students the potential for new digital services to support the transition to university, but also aim to understand the potential unintended problems or ‘tensions’ that digital services may cause—the tensions that these services might bring to the student experience. We developed 12 ‘questionable concepts’, which are a specific form of design fiction developed by Vines et al. [18]. They are design visualisations, accompanied with short textual descriptions, that set out to promote critical discussion and reflection with research participants around personal experiences and future design directions. These specific questionable concepts were grounded in earlier research conducted by the authors and other published literature on student transitions to university life. We then developed a fictional welcome brochure for new students, describing a range of digital services (the questionable concepts) that might support the transition of students to university life.

**Fig. 1:**
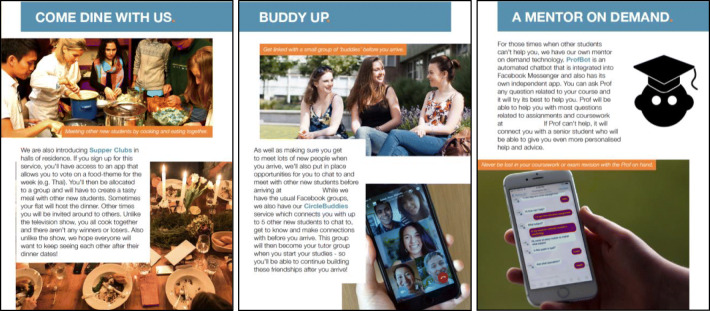
From left to right: Supper Club, Circle Buddies, ProfBot

The brochure was produced to mimic the visual communication and aesthetic of other promotional material for the university at which the research was conducted, with visuals and textual descriptions of each concept conveying what they could offer to students (see Fig. 1). We used these brochures in workshops with first year students, where they were invited to browse the available services and reflect on their value and propositions in relation to their own early weeks of arriving at university. Analysis of the workshop discussions highlighted three contexts where digital support may be influential, corresponding to the new social, home and academic environments that must be negotiated in the early weeks.

We offer three contributions to the existing literature. First, we add to current understanding about student wellbeing and the triggers for student anxiety. Second, we provide insight into the way digital interventions might impact such student problems, noting that while some systems might act to alleviate social anxiety in the classroom, others could exacerbate it. Third, we discuss the benefits and pitfalls of adopting questionable concepts as a method in this space.

## Background

### Mental health and the transition to university

Mental health problems are highly prevalent in European university student populations, particularly for those transitioning from secondary to tertiary education [19]. In part, this can be explained by new stressors: leaving family for the first time, making new friends, and facing academic hurdles. In the UK, where our research was conducted, this problem is particularly prevalent—most new students leave home to study [20]. Indeed, Universities UK, the national body for universities in the UK, produced a report in 2017 whereby the transition to university was highlighted as a key issue, calling for more explicit discussion of the mental health needs of new students and greater support during these periods [21]. They report a fivefold increase in the number of students disclosing mental health conditions between 2007 and 2018 [22]. One longitudinal study at a UK higher education institution found that psychological distress of students rose on entering university and did not return to pre-university registration levels for the duration of their course [23]. We find that students who experience mental health issues are then more likely to change courses or drop out of university [24–26]. The work of Mottiar and Quinn [27] is relevant here, who describe the transition to higher education as ‘highly stratified and complex’, noting the prevalence of failed transitions and recommends a better understanding of the ‘lived realities’ of students.

### Social media use in the transition to university

The mere perception that one’s own network is ‘ready to provide aid and assistance’ can have a stress-buffering effect during stressful life events [28]. Work by Smith [29, 30] recognises the crucial role that parents play in providing buffering social support when students move to university—offering both emotional closeness as well as encouraging independence. We already know that social support, both face to face and digital, can buffer the impact of distressing events [31]. Smith’s work explores the utility of a mobile app to connect teenage students with their parents, highlighting the importance of routine communication about challenges such as academic work, health, and finance.

People’s experiences of social technologies during times of life transition has been a major focus of social computing research [32]. There is a growing research literature that explores the way SM influences the transition to university [see 14, 33]. This is not surprising, given that SM is known to exert both positive and negative effects on young people’s wellbeing. Indeed, for low-income, first-generation university students in particular, social media interactions can support identity constructions when transitioning to a new environment by providing access to ‘mentor-like’ figures [34].

Many universities adopt or create digital social networks to support students during this transition period and help them make new friends [7, 8]. Stirling [35] describes the use of social networks (SNs) by students as an organisational as well as communicative tool in their early university encounters. An absence of peer networks are associated with early drop-out or poor student performance [10, 11]. Facebook, for example, has been noted to provide social support and improve happiness [36, 37], preserve relationships with remote family and friends [38] and help maintain offline networks [39].

Yet SM can also adversely affect wellbeing, for example when students show an over-reliance on SM exchanges with distant friends and family [40] or they experience negative responses to posts made in the new network [33], those with mental health problems can become overly dependent upon SM. Thomas et al. [16] explicitly explored the role of SM during the transition to university. They asked students to keep a record of SM interactions during arrival at their new university, using Pinterest to create a scrapbook of SM exchanges. The authors describe three identity transition processes that could be supported or hindered by SM: affirmation (strengthening their home identity), assimilation (developing a ‘host’ identity) and integration (maintaining the old identity while embracing a new identity).

Here SM played a big part in negotiating identities for students preparing to leave home, and facilitated affirmation of their existing community ties prior to starting university [16]. SM was often seen as a good way to navigate the development of friendships early on for students; however, anxieties and issues of identity were also heightened by the use of technology. Students experienced difficulties presenting their ‘authentic self’ online, partly due to issues of context collapse [41]. Recent work in this space has also touched on this notion of authenticity [42], finding that for LGBTQ+ students, SM can be used to conceal information about sexuality to avoid ‘coming out’ again when transitioning to university. Bailey et al. [43] also demonstrate the importance of authentic versus self-idealised representation on SM for improved life-satisfaction. We believe that the issue of authenticity, of presenting a ‘true’ version of yourself on SM at a time in life when you are negotiating who you want to be, is a pressing issue for students.

## Method

In this study, we sought to better understand student transition to university and the ways that digital systems and services can support or hinder this transition. We were interested in how the types of systems and services that universities may offer in the near future—or those that might exist in the future based on contemporary trends in the technology industry and education sectors—related to the transition experiences of new students, and whether they might meet their needs (or not). We were especially concerned with exploring if proposals for systems that might broker interactions between new peers might have unforeseen side effects in relation to student wellbeing, mental health and identities.

### A brochure of the university of the near future

In order to engage students in discussions around their experience of transitioning to university and relate these to potential future design spaces, we drew on prior work at the intersection of design fiction and co-design. There is a growing body of work in the field of HCI demonstrating the value of ‘design fiction’ and speculative approaches to design in developing critical dialogue with research participants, stakeholders and users around future paradigms of technology use [44–47]. One such approach for engaging research participants through design fiction is ‘questionable concepts’. Vines et al. [18] developed questionable concepts as a means to bridge ethnographic and co-design activities in their research on banking practices for ‘eighty somethings’ (people in their 80s and older). The questionable concepts were visual illustrations and short textual description of fictional designs that responded to themes and issues identified in the prior ethnographic work. However, while seemingly addressing an identified need of research participants, the questionable concepts were purposely left ill-defined in terms of how they may work in practice, and had qualities about them which inferred they might cause new problems while resolving others. In their work with the eighty somethings, Vines et al. demonstrated the ways in which questionable concepts elicited critique in group workshops; however, they also noted a myriad of ways participants used the concepts as a means to re-articulate their own values and positions on the context under enquiry, and offered alternatives to the proposals that were more in keeping with these.

In our own enquiry, there were three main motivations for adopting an approach that made use of questionable concepts. First, fictional depictions of emerging student support technologies can help to provide a more critical lens on the technologies themselves and on the values they represent [48, 49]. Second, student responses to questionable concepts could generate valuable empirical data about their assumptions and help to elicit aspects of the new student experience that we were previously unaware of. Third, as fictions, questionable concepts can be carefully constructed to tap into elements of that experience, i.e. to facilitate the research enquiry in particular ways. For us, this last point was important, as it guided us to create questionable concepts that set out to address some of the known student anxieties around the transition to university (e.g. loneliness and missing people from home, failure to make friends, not ‘fitting in’, not doing well academically and not keeping up with peers) but responded to them in unsophisticated ways that raise more questions than answers. At the same time, while being unsophisticated they still spoke to contemporary trends and developments in digital services being deployed by universities to support their students. In being ‘questionable’ in this way, and balancing a lack of sophistication with a sense of being realistic, the intention was the concepts would facilitate discussions about their appropriateness and enable discussions amongst students around potentially sensitive and personal aspects of their transition to university.

For the purposes of our study, we created 12 ‘questionable concepts’ that each spoke to issues around the transition to university. Each concept was grounded in previous work [50] which assessed the value of social media and the related ‘identity work’ undertaken by students when moving to university for the first time. The results of this previous work were discussed amongst this paper’s authors during an ideation session, with the aim of summarising the key issues—e.g. managing relationships, academic anonymity, self-presentation on SM. As a group we then began to develop ideas for near-future systems which could address some of these student concerns. With refinement, these ideas were agreed upon and became the 12 questionable concepts outlined here.

The nature of the issues identified in this prior work was diverse, and thus our questionable concepts spoke to a range of different challenges students told us they faced. For example, StorySync responded to issues international students experienced managing relationships in their home country via SM, where they often discovered their SM posts were not seen by friends because of time-zone differences. StorySync proposed a way to delay posts to social media and sync at appropriate times. Another of our designs—Pssst—responded to concerns of anonymity in class and anxieties around what other students would think of you. What Pssst offered students was an easy way to anonymously query content and ask for help. Other designs attempted to speak more directly to technology-driven agendas of various service providers whose customers are in the university sector and seek to provide more support for university students. For example, ProfBot spoke to the use of automation in providing learning support to students in place of a human academic, while Noobles reflected on the adoption of bespoke SM systems by many universities which hope to ‘broker’ relationships between students. We provide an overview of the 12 questionable concepts in Table 1.

**Table 1:** Synopsis of our twelve questionable concepts

Ambient Pillow: if your loved one also has one of these pillows, then they can be paired and they respond subtly by colour and touch as they are hugged and squeezed.	
Circle Buddies: our Circle Buddies service connects you with up to 5 other new students to chat to, get to know and make connections with before you arrive.	
CoffeeGrindr: a recommender-reward service, where you meet for a drink with other students to discuss coursework, exams, and other course content. By profiling you we make suggestions on who to meet-up with.	
Noobles: a service that makes your various social media streams and physical location more visible to others on campus. Screens in communal areas display updates from your public social media profiles.	
ProfBot: an automated chatbot that is integrated into Facebook Messenger and also has its own independent app. You can ask Prof any question related to your course and it will try its best to help you.	
Pssst: an anonymous social network for students on the same courses—where you can ask each other questions about course content and get advice about the topics you learn each week.	
Safari: tours of this fine city where you will get to visit famous landmarks, mysterious places many people do not know about.	
Similarity Index: using digital algorithms based on questions students answer before arriving at university, we can create your Similarity Index with other students. This allows students with similar cooking, music or socialising preferences to be housed together.	
StorySync: a quirky plug-in for social media services like Snapchat and Facebook to help you keep your social media stories synced to the time-zones of others.	
Supper Club: an app that allows you to vote on a food-theme for the week (e.g. Thai). You will then be allocated to a group and will have to create a tasty meal with other new students.	
TeleEats: an app that works on phones, tablets and web browsers and allows you to have a video chat with a loved one while you both follow the same recipe. You can cook your favourite meal while chatting over distance.	
YourStories: a unique story comprised of social media content. We will send you a bespoke, physical book that will be a physical memento of your time at university.	

Our 12 designs were collated in a ‘welcome’ catalogue for new university students, branded for the following academic year. In creating the catalogue we purposely used official branding schemes for the host university, to the extent that our catalogue of fictions could sit alongside other student promotional material and not be out of place. This was done to promote more genuine engagement from participants around the implications that these services would have for future students, posing to them questions such as: What if these services had been introduced for you this year?; How do these reflect the experiences you had when joining the university? and How might these new services affect the experiences of new students? We anticipated that some of the fictions would be critically received, and as such saw them as a means for opening up new design spaces, to elicit nuanced discussions around experiences, and to reframe the problem space for designing for student transition to university.

### Participants

Participants were recruited from a large UK university, and were all first year undergraduates. Students were recruited in a number of ways—word of mouth, posters around campus, and announcements at the start of lectures—but predominantly through a university-wide e-mail to all first year students. As a result, participants were studying a variety of courses, living in different university accommodation, and came from the UK as well as Libya, Norway, Poland, Jakarta and the Czech Republic. In total 32 first year students took part (8 male, 24 female) with a mean age of 18.8 years (S.D. 0.7 years). The workshops took place in November, meaning all of the student participants had been at the university for around 8 weeks. We acknowledge that our mean participant age is reflective of a ‘typical’ undergraduate student, but note that we did not intentionally omit mature students—though some of the questionable concepts were more relevant for this younger cohort.

### Procedure

We conducted two half-day (approx. 3 h) long workshops on university campus. The students participated in two large groups, which were then split up into smaller groups of 4–5, each with a facilitator (a member of staff working on this project) to guide discussion. Following a briefing and an ethics consent procedure, students were first asked to talk generally about their experiences at university so far, responding to anonymised quotes from previous undergraduate interviews on the topic of induction and settling in. This was done to help participants relax into the group discussion, and to start to engage with topics of discussion related to the research without necessarily being explicitly asked to speak about personal experiences. After this, students were each given the catalogue, and asked to read through it. Students were asked to review each questionable concept in turn, and explain how appropriate or feasible they felt these would be for new students, as well as their own perception of them. Such group discussions have been shown to be valuable in eliciting sensitive and novel forms of data [51]. Each group had a facilitator who prompted critical discussion of the various designs but encouraged the students to support their views by drawing from their own experiences. There was an audio recorder on each table, and the facilitators took photographs to document the process. Participants were paid £20 each for their participation. The study was carried out in accordance with the recommendations of Northumbria University Ethics Committee with written informed consent from all participants.

### Analysis

Transcribed data was analysed with the aid of QSR International NVivo software. Following Braun and Clarke [52], it was first important to familiarise ourselves with the data, which meant the lead researcher read and re-read the typed transcripts. This generated initial codes through constant comparisons between the data, and allowed them to search for key themes, whereby patterns and repetition emerged in the data. We followed the advice of Guest, MacQueen, and Namey [53] by monitoring closely themes throughout the process. We found this had the advantage of endowing flexibility and was well suited to our dataset, which was relatively large and was shared between two authors to monitor coding.

Following acknowledged practice in qualitative methods [52], two of the three research team discussed codes generated by the lead researcher (who used open coding with no a priori framework). Discrepancies (usually over framing of codes) were reconciled with re-review of the broader theme structure, and a re-labelling of themes where needed. The third author then checked the analysis agreed by the two other authors. This ‘researcher triangulation’ [54] led to the themes described in the paper.

## Results

We identified key concerns running through our data around social and performance anxiety and we were also able to map out design elements that exacerbated these anxieties. From our analysis, we have been able to identify more carefully what gives rise to these types of student anxiety and the specific contexts associated with them. We group these themes in terms of the stressors student face with social bonding (facilitating connections and making friends), in their home (privacy and shame in communal living) and academic environments (social comparison and academic support), showing how technologies can ameliorate or exacerbate problems. In our discussion, we also consider the benefits of using questionable concepts in this space.

### Facilitating connections and making friends

Critical for new students is the anxiety associated with making friends in their new environment. This was especially a concern in relation to the perceived unknowns of moving to university, with students often concerned about who they may be living with, who will be on their course, and whether they will be able to get on with them. Unsurprisingly, social media was felt to have the potential to both alleviate and exacerbate these problems. As in prior work [e.g. 16], participants explained to us how they would use Facebook groups and other SM to get a sense of who the other new students might be. Participants also recollected the ways they curated and edited their social media profiles to ensure pictures and content they felt would not show them in their best light to new flatmates, friends and peers:

*M5: I did clear every ugly photo of myself before I came to university, I just know how judgmental people can be and you can soon feel exposed and vulnerable if you let people see […] all those childhood photos of you.*

As well as carefully editing and removing content from their profiles, several participants made use of group chats on SM that were facilitated by their new university to link new students up before starting their studies:

*F1*^1^*: I met with one of the group chats, I don’t know which one it was in I met a couple of friends through it and we all met up before university and then when we got to university we kind of new each other so we went out on the first day.*

*M3: The thought of moving in with strangers was freaking me out because I thought what if they don’t like me, what if they hate me, to be able to talk to someone online made me feel a lot more comfortable.*

In the welcome catalogue the Circle Buddies concept was designed to further explore these issues. Circle Buddies was envisioned as a mobile application that would assist students in making connections with other ‘similar’ students—for example those on the same course, or perhaps with the same interests—prior to physically arriving at university. It would enable group video ‘chats’ with up to 5 other students, and was positioned as a simple but effective way for students to get to know each other before arriving at university, without the stresses and concerns about how they would be perceived by others originating from SM [16]. This group would then form the basis of a lasting tutor group to support students throughout their time at university. The justification for Circle Buddies seemed quite straightforward. In addition to supporting friendship making, peer support has been shown to act as a buffer against maladaptive behaviours in students [55], while working together in groups helps students form relationships with one another [56], builds trust and a support structure and reduces student anxiety [57]. Small group collaboration allows students to socialise as well as learn, and enables them to ask questions about problems [58].

Circle Buddies did help provoke discussion around the challenges of meeting new people, but raised new issues around video as an acceptable format for social engagement with strangers. We can see this in a dialogue between three female participants:

*F18: It seems like a good concept but I’m just not sure about how many people would be willing to like - would it be a video chat? […] Because yeah - I feel like, people wouldn’t feel very confident doing that with people they have never met.*

*F19: It would be a bit awkward because you might not know what to say, I think it is easier face to face.*

*F18: If there is like one person up for a video chat and no one else - it would be a bit awkward.*

While a small number of participants saw the potential value of Circle Buddies—especially if ‘*there were options to find common interests or what I would learn from them*’ (M2)—the resounding response was more critical. Many participants referred to the potential for such video chats to be ‘awkward’, and instead felt that in person, face to face, meetings would be more appropriate. There was a sense that video would be too personal, too intimate, to facilitate discussion between strangers: ‘*Its quite personal, if you don’t know them - cause its not on like a professional level either like making friends*’ (F9). But more importantly, systems like Circle Buddies were viewed as an attempt to engineer relationships and friendships for students on their behalf: ‘*I think its a balance cause you don’t wanna force friendships - and then its just awkward for everyone involved*’ (F22). Participants talked of many instances of not getting along with other new students, and not wanting to be around those people if it was not essential. Circle Buddies was perceived by some as just another way to enforce friendships that might not work, akin to placing people in halls of residence with flatmates they did not get on with (which we elaborate further on below):

*M5: I know some people would probably sign up, but it’s not for everyone - probably before and most of the time you sign up to something to talk to random people um you talk to them, you know them before, and then when they meet you in person, they might dislike you […] they might decide I don’t actually like you so, let’s say all four of them don’t like you, or you don’t like each other, but you’re all nicey-nicey on this but you have to see them, so it’s one of those things where you’re being forced to meet people you don’t like at all.*

Several participants reflected on concepts like Circle Buddies in relation to experience of group work on their courses. Often it was felt that groups would be assigned at ‘random’, and the perception was that applications like Circle Buddies would be equally unsophisticated in linking new students together. But it was also viewed as a way of removing some control from students in forming their own connections and learning about who they wanted to spend time with at university. A further concerns was that such overt attempts to link students together based on preferences or some identified shared interests and qualities would end up creating new ‘filter bubbles’ and limiting the range of new people they might be exposed to:

*M4: I dunno, before uni I’d say it’d be a good idea, but like of my flatmates we’re all completely different like so and we’ll all still get along really well.*

*M6: I think you should mix with people who are different to you, so you get new ideas and new people to do new activities to help you grow as a person, and I think if you’re all together and you’re the same type of person, it’s basically just like shouting into an echo chamber, you don’t get much back.*

In designing a ‘questionable concept’ with the aim of removing the effort required to make friendships early on, a number of design issues became obvious. Firstly, video chat is not only intrusive, but can be perceived as forced, awkward and unnecessary. Secondly, digital befriending carries risks of creating obligations to people you may come to dislike and thirdly, it carries the risk of filtering out interesting new relationships. In short, digital befriending services may not be ‘transformative’ unless the social aspects of the engagement are fully considered.

### Privacy and shame in communal living

Several of our concepts explored aspects of communal living in student accommodation and halls of residence. Indeed, some of our participants explained how the communal spaces in their flats were critical to feeling socially connected and provided important opportunities to spend time with flatmates:

*F25: We can all sit in the kitchen and cook and eat together and things like that […] we’ve got a TV in our kitchen but no one has one in their room so if we ever want to watch things we’ll all watch it together but not many of us go out that much so its kind of like its more of a - its almost a comforting thing where at home you could just pop downstairs and see your parents things like that its almost like you can pop into your kitchen and see someone and have a conversation so it is quite nice. It stops you from being this lonely.*

One of our concepts—Supper Club—explored communal eating further, looking at its potential for building and cementing relationships. It was based upon the possibility of using common interests in cooking and eating to form new social bonds. Supper Club was explicitly intended to support students’ social needs, recognising again that they do not easily make friends outside their immediate flatmates and noting previous work where students said that they typically bonded over food and cooking [16]. Supper Club would be facilitated by a smartphone application where students could vote every week on a food-theme (e.g. Thai), be allocated to a cooking group, and then cook with other students in a communal kitchen in one students’ flat. The description explained that sometimes students would have to ‘host’ the event, but at other times they would be invited to another student’s accommodation. In conceiving of Supper Club, an assumption we also made was that, for new students, the spaces for food preparation and communal eating often become the focus of interaction within their accommodation. Indeed, the notion of cooking together in order to promote friendship and well-being has been adopted in a number of different contexts, for example with firefighters [59] and offenders [60]. In both instances this change resulted in commensality and cemented friendships.

Some aspects of Supper Club were appreciated. For example, students liked the focus on cooking, in part because it reminded them of being at home with friends and family, something Bales and Lindley [50] emphasised is important for new students. Indeed, one student in our study explained that they had already tried to arrange a cooking event, with a focus on international students:

*M8: The Business School had a competition about how to improve first year’s life and we did something very similar, like the flats and like our goals is to do a big food fest with different nationalities.*

However, as discussions surrounding the concept delved deeper into the feasibility of doing it, students started to raise stressors that might prevent their participation. This was, in part, related to how participants felt they were very limited in their cooking skills:

*F13: Now I’m still getting used to cooking and it was really stressful for me in the first three or four weeks I just didn’t know what to cook I was calling her [Mum] and I was just asking what to do.*

The time-dependant nature of Supper Club also highlighted problems with scheduling for students. Living with people on different courses meant differing timetables, so group activities such as cooking together were restrictive:

*F24: Everyone normally cooks for themselves, especially when you’re in different lecture times. We only really sat down properly during Freshers week whereas now we’ve just come back at different times […] Everyone cooks their own stuff.*

Perhaps most critically, however, Supper Club was also problematic because of students’ living arrangements in first year. In discussing participating in the service, students were very vocal about their dissatisfaction with their flats and houses, and specifically the cleanliness of their shared spaces:

*F14: I really like the idea of cooking together but I can’t imagine hosting anything. I know like me and two of the friends we are close but you know like, the kitchen is so messy its horrible so I can’t imagine bringing other people to our kitchen because sometimes it happens if we are not like getting on well with our flatmates, so I think it would be better to be in someone else’s kitchen, or maybe like some space in uni because I cannot imagine to invite people into our flat it is so horrible.*

*F24: I know some people who live with really messy flatmates and they’re just fed up cause they’re always cleaning all the time and it’s just really dirty. So I think it can divide people.*

Another related concept in our catalogue was TeleEats, an application that enabled students to have a video chat with a loved one (living elsewhere) while they both followed the same recipe. This follows work by Smith [29] who proposed digital solutions which encourage students to remotely participate in family rituals, such as dinner conversations. TeleEats enabled exactly that kind of interaction and many students liked the idea of learning about new cuisines and acquiring new cooking skills. It also, therefore, acknowledges the problems F13 noted above about not knowing how to cook for themselves.

TeleEats was received well, firstly in terms of staying in touch with family and friends back home:

*M5: That’d be good as well, talk to your parents, and they’ll help you, I dunno like how to cut like a carrot or something, and you’ve got the recipe there you can ask your mum how you do this properly.*

*F5: Cause they’ll get more sort of excited about cooking knowing at the same time they’ll get to talk to their family they don’t see a lot it’ll make them want to cook instead of just ordering a kebab.*

Once again, however, the lack of available ‘safe spaces’ to use for cooking was a problem and the anxieties associated with communal living reappeared:

*F14: I think it is actually a good idea but the problem is that I still can’t imagine today like doing it in the kitchen, because there are people like coming and - if I was doing it with my mum I would like it to be private you know - so if I was living alone and my kitchen looked good I think it would be nice yeah.*

Overall, our concepts opened up discussions around how the student home was not generally viewed as a comfortable space. Despite most of the students having a shared kitchen and an area in their halls of residence specifically designed to bring them together, it was felt that these spaces could not reasonably be used for group social activities. Research has demonstrated that satisfaction with communal areas is a significant predictor of general satisfaction with university accommodation [61], with other work suggesting university accommodation can generate stress [62]. Here we see it impacting on student’s emotional investment in communal activities. The questionable concepts raised fundamental concerns about both the physical communal living environment and the new social connections associated with moving in together.

### Social comparison and academic support

An issue throughout the workshops was the ways students would compare themselves to other new students. The careful construction of social media profiles, referred to earlier, was done in part as a comparison to the content shared on other new students profiles. Comparisons with other students also permeated the learning experience, with many of our students worried about their own ability in comparison to others on the course. This manifest in various ways, but an often referred to concern was they felt that asking questions in lectures was not easy:

*F5: ‘If there is something I don’t get I would either be too scared to ask the lecturer in case I seemed too stupid, or a lot of the lecturers at the end like are too busy, they just sort of leave straight away you never get a chance to ask them.’*

*F22: Quite a lot of people on my course did business or economics at a-level and I didn’t so some of the stuff we’ve been doing I didn’t understand it so I facetimed my dad cause he’s in business so he’s been trying to help me - I just need someone else there to talk to.*

They agreed that it was easier to turn to each other for support. Sometimes this was to avoid having to ‘expose their ignorance’ to lecturers. Others said it was simply because lecturers were busy or hard to pin down. It also meant confiding in a small group of closer course mates rather than opening up to the whole of their course. The net result was that many had proactively self-organised in order to support each other with academic problems:

*F1: We decided to make a group chat about like, with all of us in from our course because our lecturers weren’t helping and we were getting re-directed everywhere and all the lecturers were saying different things.*

*F2: Yeah my tutor travels a lot because she teaches internationally so to get in touch with her its a nightmare.*

Although peer support seems like a natural response, particularly for those students who experience social anxiety or low self-esteem, it raises problems in terms of the quality of information and advice circulating in the group. In response to these issues, we explored different approaches to student mentoring [63] and introduced ProfBot as a ‘mentor on demand’. This chatbot, integrated into Facebook Messenger, would be able to answer questions about academic issues such as confusion over assignments or specific aspects of coursework. In the event that ProfBot could not help, it had a feature that could connect students to a more senior student at their university to help.

Some students appreciated this concept and perceived ProfBot as a way of providing a reliable source of information and guidance with coursework. In particular, ProfBot was seen as a possible means of alleviating the social anxiety students experience when asking questions in class. As a concept it facilitated open discussion about the anxieties associated with public speaking, nervousness at asking questions in class, and a preference for one-to-one interactions over group discussion. Our students were keen to find some kind of support whereby they could ask questions or seek clarification of points raised in class anonymously or without fear of judgement:

*M3: People struggling to begin with [would find this useful], I think first year students definitely and getting through their first couple of assignments, if they’re not sure about something and they’re too shy to go and speak to the lecturer then have a go at this.*

ProfBot was contrasted with reactions to Pssst, a concept which allowed students to anonymously ask questions in real-time to other students in relation to the content of a lecture. The latter was heavily critiqued, with concerns raised about the moderation of content and quality control on responses to questions. Furthermore, it was felt that while it was hard to ask questions in the moment, it was also because questions and clarifications are often needed outside of the lecture theatre when coursework and assignments were being worked on. Because of this, ProfBot, with its always available ‘mentor on demand’ was felt to be a superior proposition:

*F24: I’d find this really helpful. Cause like - you just can’t contact your tutors or anything, on my course it’s just very- one of the lecturers he just hasn’t really described anything to us and on the group chat we were all like what is going on? We didn’t find out our personal tutors ‘til last week and I was going through some stuff and I didn’t understand what was going on and I really needed someone to help me, so that would be really good.*

*F5: I think something like this would be really helpful - especially if you’re like doing something dead late at night you can’t ring them or go into uni.*

However, a number of students questioned the trust and reliability of ProfBot:

*F9: It’s a good idea but I think my only concern would be like would they understand what you’re saying or would they be giving you the help you actually needed, would it be specific to what your issue was? I dunno, I think it’s a good idea if it worked well it would be really good.*

*F18: I’d probably double check whatever it told me before I put it into like an essay or something - so like small questions I think it would be quite useful.*

Note, in this last comment, the assumption the ProfBot would provide specific answers to questions, rather than scaffold learning that would take place elsewhere. In other words, students were prone to think of ProfBot in terms of ‘spoonfeeding’. Our design thus promoted open discussion about the reliability of information on offer, but also opened up discussion of the ways in which the lived experiences of students (involving late submissions, reliance on ready answers, elusiveness of lecturers, fear of failure and low self-esteem) drove them towards their peers in their information seeking behaviour.

## Discussion

In our analysis of student responses to the brochure of questionable concepts, we captured many of the challenges facing new students in their academic, social and home lives. In the following discussion sections we draw out further insights across our data in relation to what is already known about the undergraduate student experience. We then go on to reflect more carefully on the value of ‘questionable concepts’ as a method in this context, especially in terms of the way such provocations can prompt highly personal open discussion around sensitive issues.

### Designing for positive student transitions

#### Reducing social anxieties without social engineering

It is apparent that social anxiety can be quite acute for new students and indeed has been recognised as one of the most common mental health problems for students as a whole [64]. A finding in our work was that our students tended to reject interventions (digital or otherwise) to help them establish friendships before they arrived at university. While we had an expectation that students would prefer a social connection to those they had already met, or those who were deemed similar to themselves, we find that students here claimed they preferred to mix with new and different people. They were cynical of attempts to engineer relationships based on preferences and attitudes and highly critical of certain technologies (such as video calling) that would attempt to unite strangers which would leave them feeling embarrassed and awkward. Such digitally mediated introductions do not therefore offer a means of reducing the social anxieties associated with friendship formation but may even exacerbate them. This may be in part because students have demonstrated they can form their own networks prior to arriving on campus [e.g. 65], reducing the need for such engineering.

#### Negotiating the rules for communal living

A surprise for us was the stress associated with a new home environment, where we uncovered issues around privacy and the negotiation of living standards. Thus, the TeleEats concept, while praised for its ability to connect students with their family and friends by cooking together, became problematic when students realised they would only be able to access it in communal spaces. Similarly, Supper Club was appreciated as a means to get people engaged in social activities within halls of residence, but students could not emotionally invest in these schemes. Prior research has highlighted how the communal student kitchen is a setting for great social activity around food [66] and a highly dynamic place where students manage boundaries between their private and social lives [67]. Because of the sociality inherent in our concepts they were first deemed valuable. But viability was limited because of the dissatisfaction with communal living, and an uncertainty surrounding the negotiation of shared use of space. A number of papers discuss the implications of student accommodation design and have identified poor quality of accommodation as a major concern for students [68]. Amole [69], for example, found that more than half of their 1124 student participants were dissatisfied with their residences, and in particular, the social qualities of their residences—the kitchenette and bathroom. Thomsen and Eikemo [70] found, unsurprisingly, that students prefer individual facilities such as bathrooms and kitchens when choosing communal living spaces. Yet such papers do not capture the social dynamics of student housing and the ways that interpersonal dynamics can act to inhibit social exchange more broadly.

#### Scaffolding learning with automated systems

Additional issues around performance anxiety emerged in response to our fictional systems offering new ways to interact with lecturers. Many students are loathe to speak up and raise issues when they have academic concerns. Our participants displayed some enthusiasm for ideas like ProfBot, as they appreciated the opportunity to ask questions without the social embarrassment of putting a hand up in class, as well as the chance to seek guidance out of office hours. In fact, we were shocked at the ways the students were seemingly content with relying on such an automated system to provide timely and reliable information. However, for some, trust issues were prevalent and students expressed concern that they would still need to check ProfBot answers with a member of staff, because they could not verify its accuracy—trust in the system was minimal. While ProfBot was fictional, there are increasing examples of chatbots been used to support student access to services (as at Lancaster University in the UK [71], or acting as a course companion that engages students in ‘conversational learning’ [72]). The reactions from our participants suggest we should be cautious around the promises of such technologies and avoid the perception that they can be relied on at any time, for any question. Instead, learning from the suggestions of a smaller number of our participants, we might suggest such bots act to scaffold learning, by steering students to search for their own resources elsewhere, providing hints and tips rather than direct answers to questions that will unlikely be very clear.

### Reflecting on the use of questionable concepts

In earlier research in HCI, richly elaborated scenarios, populated with fictional characters [48] or highly ambiguous fictional scenarios populated with realistic, well rounded and richly depicted characters [45] have been used effectively to elicit lived experiences from participants. Such ‘fictional’ techniques overcome some of the dangers of presenting existing artefacts to potential users, given that these can constrain thinking about alternatives and inhibit the imagination [73].

Drawing on one such approach that makes use of fictions—questionable concepts—we found our catalogue to be effective in eliciting reflection on a range of personal circumstances. Our students spoke very honestly about their difficulties and were relatively unconstrained in the way they would describe the horrors that might result from strangers visiting their dirty apartment or the anxieties they might feel in simply asking a question in class. We were certainly able to elicit the ‘felt lives’ [74] of the students in ways we had been unable to achieve previously through more traditional interview-based approaches to data gathering [16]. This was, in part, because the concepts themselves acted as tickets to talk, to stimulate discussion around what was wrong and right with them. However they also enabled us to bring the focus of discussion to specific situations, settings and moments that have meaning for students, acting as a starting point for revealing personal experiences and circumstances that problematised the ideals the concepts presented.

Some of the questionable concepts worked very well, eliciting what appeared to us as honest and open responses. Others were far less successful however. In general, those that were simply agreeable (like Safari) but unexciting were uninformative, and offered little empirical insight of value to the research (hence not being reported on in the findings). Those such as Noobles that contained purposely problematic elements were effective in provoking discussion about the shame of having personal lives (as recorded on SM) exposed to strangers via a public display. But perhaps the most valuable questionable concepts were those that seemed, initially, to be relatively ‘safe’ to the designers, but were deemed problematic by the students. This included the social apps (Supper Club, Circle Buddies) that uncovered new dimensions to the supposedly simple act of making new friends at university. In these cases the concepts were not taken as well-rounded solutions to a problem; rather the students used the concepts as an opportunity to rearticulate and reorient the problem space.

This is not to say the social applications did not receive criticism. Many of these concepts were based on earlier work in which students talked about the social pressures associated with existing social media sources [16]. By presenting our questionable concepts as ‘new’ digital services to be provided by the university, we hoped students would be able to consider them as distinctly different from the social media platforms they may be so familiar with. Yet often students dismissed our propositions as irrelevant, arguing that existing social media platforms (usually Facebook or Skype) would be sufficient. They asked why we would expect people to learn a new digital system and showed reluctance to ‘move’ away from a familiar social media platform to a new one.

*F8: It could be a good idea but I don’t know why I’d need an app for it. You could just have a Facebook group and have a poll there [for Supper Club]- […] it’s not a good idea to have people move social media.*

Similarly, when talking about ProfBot, students could make connections between systems they already had in place to speak to other students to get advice.

*F19: We have like a seminar group chat - we ask a lot of questions to each other on it - and then there is a whole business management group chat - with like everyone on it as well so now and again people ask stuff on there.*

This kind of digital support is not unusual at university, and instant messaging services can play a positive role in academic achievement and student satisfaction [75]. However, many institutions like to create their own ‘mini platforms’ that replicate key features on other commonplace platforms, such as chat systems and forums. Our participants spoke critically about existing platforms provided by the host institution such as ‘Campus Society’ that were deemed redundant and consequently showed poor uptake.

*F19: It’s like an app where people from the same university can chat and post things and get to know each other, about your course and whatever - it is a way of finding people from your university. I downloaded it in the summer holidays before I came to university to see if I could find anybody but there was nothing really there.*

In their prior work on questionable concepts, Vines et al. [18] highlighted the ways in which critique of concepts from participants can be harnessed creatively, to generate sophisticated commentaries on technology and lead to stronger design concepts. In this case, however, the criticisms often tended to close off discussions, with the concepts considered as ill-thought-through attempts to replicate the features and functionalities of existing systems. This was, at times, because the critical intents of the concepts, as both a commentary on and alternative to the dominant social media systems, were too subtly communicated in the catalogue. At the same time, however, their criticisms could be read as a general criticism of designing and deploying technology for the sake of technology. Notably, while their critique often closed down discussions, they did include examples of other technologies that could be resourcefully reconfigured and redeployed to enable the types of experiences our concepts set out to support.

## Conclusion

We designed a brochure of questionable concepts to explore the transition to university for new students. The issues we unveiled reflect a broader spectrum of triggers for student anxiety than is typically considered in student research, where most work has a focus on academic performance. Given current concerns around mental well-being and dropout at university [76], it is surprising that such limited attention has been given to the full range of challenges for new students. In addition, note that recruitment took place prior to the Covid-19 pandemic. We anticipate that the anxieties surrounding the increased use of digital platforms during this pandemic will have only served to exacerbate the student issues we highlight here. These findings support our earlier work in describing the complex challenges for students during the transition period [16] and highlight some of the limitations of digital support in this space. Our questionable concepts were generally effective as provocations for student discussion around the many social and performance anxieties associated with transition, also highlighting the challenges in successfully negotiating communal living arrangements, which we would see as an interesting area for development.

## Data Availability

Due to the sensitive nature of this research, participants did not agree for their data to be shared publicly, so supporting data is not available.

## Footnotes

1 F/M denotes female/male, followed by participant number.
